# Grape Marc as a Functional Feed Ingredient for Farmed Snails *(Cornu aspersum maximum*): Effects on Production Performance, Parasitological Status, and Meat Quality

**DOI:** 10.3390/ani15182680

**Published:** 2025-09-13

**Authors:** Marianthi Hatziioannou, Alexandros Theodorou, Konstantinos Apostolou, Efkarpia Kougiagka, Persephoni Giannouli, Ioannis T. Karapanagiotidis, Smaragda Sotiraki, Athanasios Exadactylos

**Affiliations:** 1 Department of Ichthyology and Aquatic Environment (DIAE), School of Agricultural Sciences, University of Thessaly (Uth), Fytokou Str., 38446 Volos, Greece; altheodo@uth.gr (A.T.); apostolou@uth.gr (K.A.); ekougiagka@uth.gr (E.K.); ikarapan@uth.gr (I.T.K.); exadact@uth.gr (A.E.); 2 Department of Biochemistry and Biotechnology, School of Health Sciences, University of Thessaly (Uth), Viopolis, 41500 Larissa, Greece; pergian@uth.gr; 3 Veterinary Research Institute, Hellenic Agricultural Organisation (ELGO)-DIMITRA, 57001 Thermi, Greece; sotiraki@vri.gr

**Keywords:** snail nutrition, grape marc, snail parasites, meat quality, chemical composition

## Abstract

**Simple Summary:**

As more consumers include snail meat in their diets, ensuring higher quality standards is becoming increasingly important. To support welfare, growth and improve the quality of snail meat, farmers are looking for sustainable ingredients to add to snail feed. This study tested the use of grape marc (GM), a by-product from winemaking that is rich in antioxidants, as a natural supplement in the diet of farmed snails. We fed snails with different amounts of GM (0%, 7% and 14%) and recorded how it affected their growth, health, and meat quality. The grace marc did not affect the growth performance but lowered the number of parasite eggs in the feces. The feed enrichment improved the tenderness of boiled fillets without changes in color. These findings suggest that GM can be a useful, natural addition to snail feed that may improve both animal health and the quality of the final product.

**Abstract:**

In countries where edible snails are a valued food source, improving snail meat quality is a priority. The aim of this study was to evaluate the effects of grape marc (GM) supplementation in the diet of *Cornu aspersum maximum*, relative to production performance, animal health and quality of snail meat. GM was added in feed for snails at an inclusion rate of 0%, 7% and 14%. The results demonstrated that GM supplementation can be included in the diet of farmed snails, resulting in a slight increase in feed intake without compromising growth performance. The inclusion of GM in the diet reduces from 2480 EPG (parasites’ eggs per gram of feces) after a non-enriched diet, compared to 700 EPG in the feces of snail fed supplemented diets. Nutritionally, the highest dry matter (24.14%) was observed after 14% feed enrichment. Feed enrichment improved the tenderness of boiled fillets (close to 6 N), with no color changes. Enriched diet may lead to the production of functional food which fulfils consumers’ demands for high-quality products. Notably, this was the first study that used GM as a feed component in heliciculture, highlighting its potential as an alternative and sustainable dietary source.

## 1. Introduction

In recent years, there has been growing interest in incorporating natural antioxidants into the diets of farmed animals, as well as in meat products. Among these, grape marc (GM), a by-product of the winemaking process that includes skins, seeds, stalks, and stems left after grape pressing has been increasingly explored for its antioxidant properties in animal nutrition. It is estimated that approximately 20% of the total grape weight used for winemaking results in GM. Given the high wine production in Mediterranean regions, such by-products are abundantly available [1]. Despite its availability, the nutritional application of GM in animal feed remains limited, primarily due to its high fiber content, low protein and energy density, and the presence of anti-nutritional factors such as polyphenols and tannins [2]. However, GΜ is rich in bioactive compounds, which, according to in vivo and in vitro studies conducted in recent years, exhibit antioxidant and antimicrobial activity [3,4,5]. The use of polyphenols is recommended to limit lipid peroxidation and preserve animal health and product quality [6]. Lipid peroxidation is a matter of great interest, both for the food industry and for consumers, as it leads to the development of unpleasant smells and tastes in food products. The applications of winery by-products in livestock nutrition are very significant because of their antimicrobial and coloring properties affecting the shelf-life and quality of the product [7,8]. Winery by-products, such as grape pomace, grape seed flour and GM, are cheap and highly available, having been used recently in the nutrition of many farmed animals. In view of the wide availability, low cost, and high fiber concentration of this by-product [9], its use could help to reduce production costs. Nevertheless, most existing research has focused on terrestrial livestock, while there is a notable lack of studies regarding farmed invertebrates, particularly mollusks. A rare exception is the work of Bullon et al. (2025) [10], who demonstrated that dietary inclusion of GM in abalone significantly enhanced growth performance. These preliminary findings suggest that GM may serve as a sustainable and functional feed additive in aquaculture systems.

Heliciculture, or snail farming, is widely practiced in various regions including West Africa, Europe, and Southeast Asia, with its growing economic relevance documented in both rural development and sustainable protein production [11,12,13]. For many years, edible snails have been an integral part of the culinary tradition in various European countries. Among these species, *Cornu aspersum maximum* (Gros Gris in French) occupies a prominent position, as its farming is widely established. In countries where edible snails are a valued food source, improving snail meat quality is a priority. Although significant research has been conducted on dietary formulations that enhance the growth and reproduction of snails [14,15], a knowledge gap remains in the utilization of agro-industrial by-products within the framework of a circular economy.

Previous surveys have reported the presence of parasitic infections, caused by ciliates, nematodes, and mites, in snails under rearing conditions [16]. These pests pose a significant risk to heliciculture, as they may spread throughout the farmed snail population, particularly under overcrowded condition [17]. Invertebrate immunity is intimately linked to nutritional status, and alterations in diet composition could either impair or enhance the snail’s physiological defense mechanisms. [18]

Snail meat is a source of protein and essential nutrients for the human body such as vitamins (vitamin A, vitamin E, vitamins B1, B2, B3, and B6) and minerals, especially calcium, potassium, sodium and trace elements such as iron and selenium [19,20]. Milinsk et al., 2006 reported high contents of palmitic and linoleic fatty acid in the edible tissue of *Cornu aspersum maximum* after the 3% feed supplementation with canola, soybean, flaxseed, sunflower, maize, and rice oil [21]. Also, flaxseed oil enhanced the snail edible tissue in linoleic fatty acid. Finally, pH value of snail edible tissue was estimated at 7.46 ± 0.12 by Zymantinie et al., 2006 [22].

There are few studies which relate the organoleptic and textural characteristics of edible snail tissues. Schubring & Meyer [23] noticed significant differences among the hardness values of the processed snails without shell of the species *Helix aspersa* and *Achatina fulica* compared to snail species *Helix pomatia* and *Helix lucorum*. On the contrary, the sensory panel did not express a preference for a particular species. According to Kougiagka et al., 2022a [24], cylindrical parts of heat-treated snail fillets, as foot-head tissue of snails is labelled, are more appropriate for textural assessment and high total nitrogen content, and low carbohydrate content were associated with less tenderness. Color as a factor affecting consumers was studied for fresh and processed snails. Edible tissue of fresh wild snails of the species *Cornu aspersum maximum* presented high values of L* lightness (54.67 ± 1.85) and b* yellowness (19.49 ± 1.53) and low value of a* redness (−0.03 ± 0.89). On the contrary, minced processed snails of the same species reported the lowest value of lightness (30.98) and the highest value of redness (4.02) among the species *H. pomatia*, *H.lucorum*, and *A.fulica.*

This study aims to evaluate the effects of dietary supplementation with GM at two inclusion levels in farmed snails (*Cornu aspersum maximum*), focusing on feed intake, growth performance, parasitic load, and key meat quality parameters, including chemical composition, tenderness, and coloration. The research is framed within the broader context of circular economy and sustainable animal production, exploring the potential of agro-industrial by-products as functional feed components in heliciculture. Beyond improving production efficiency and meat quality, the study also seeks to reduce the environmental footprint of snail farming through innovative feed management strategies.

## 2. Materials and Methods

### 2.1. Experimental Diets

The GM flour used in this study was produced from red grape varieties (50% Merlot and 50% Syrah), sourced from a winery located in Central Greece (Larissa, Thessaly, Greece) after completion of alcoholic fermentation. The fresh material was rinsed with deionized water to remove residual must and then stored at –20 °C until further processing. In the laboratory the GM underwent mild distillation for 15 min at approximately 94 °C to remove residual ethanol [25]. Subsequently, it was dried under controlled conditions (maximum 94 °C for 24 h). After drying, the solid substrate was subjected to manual and mechanical sorting to remove stems and leaf debris, ensuring the purity and uniformity of the sample. The cleaned and dried material was then ground using a laboratory mill, and the resulting flour was stolen through a 0.5 mm mesh to reduce the content of crude fiber and obtain a uniform particle size [26]. The final product was characterized by high dry matter content (83%), crude protein (13.78%), and crude fat (7.05%) (Table 1).

Based on the literature and pre-feeding observations, experimental diets were formulated to contain 0% (CF0), 7% (CF7), and 14% (CF14) GM flour, considered to be adequate due to high amount of fiber [25,26,27]. The composition of the experimental diets for *Cornu aspersum maximum* snails is presented in Table 1.

In our experiment, the control diet CF0 was a complete compound broiler chick feed (poultry feed for the first age stage from 1 to 30 days), manufactured by a feed industry *(Tziatzias, Single-Member I.K.E., Trikala, Greece).* The form of this feed is powdery, and the raw materials used are cornmeal, soybean meal, soybean protein, soybean oil, wheat bran, powdered milk substrate, and yeast forage. The detailed chemical composition of the feed is given in Table 2. For the purposes of the experiment, calcium carbonate (CaCO_3_) was offered *ad libidum* to the snails in a separate feeder, distinct from the main diet.

### 2.2. Experimental Snails and Culture

**Figure 1:**
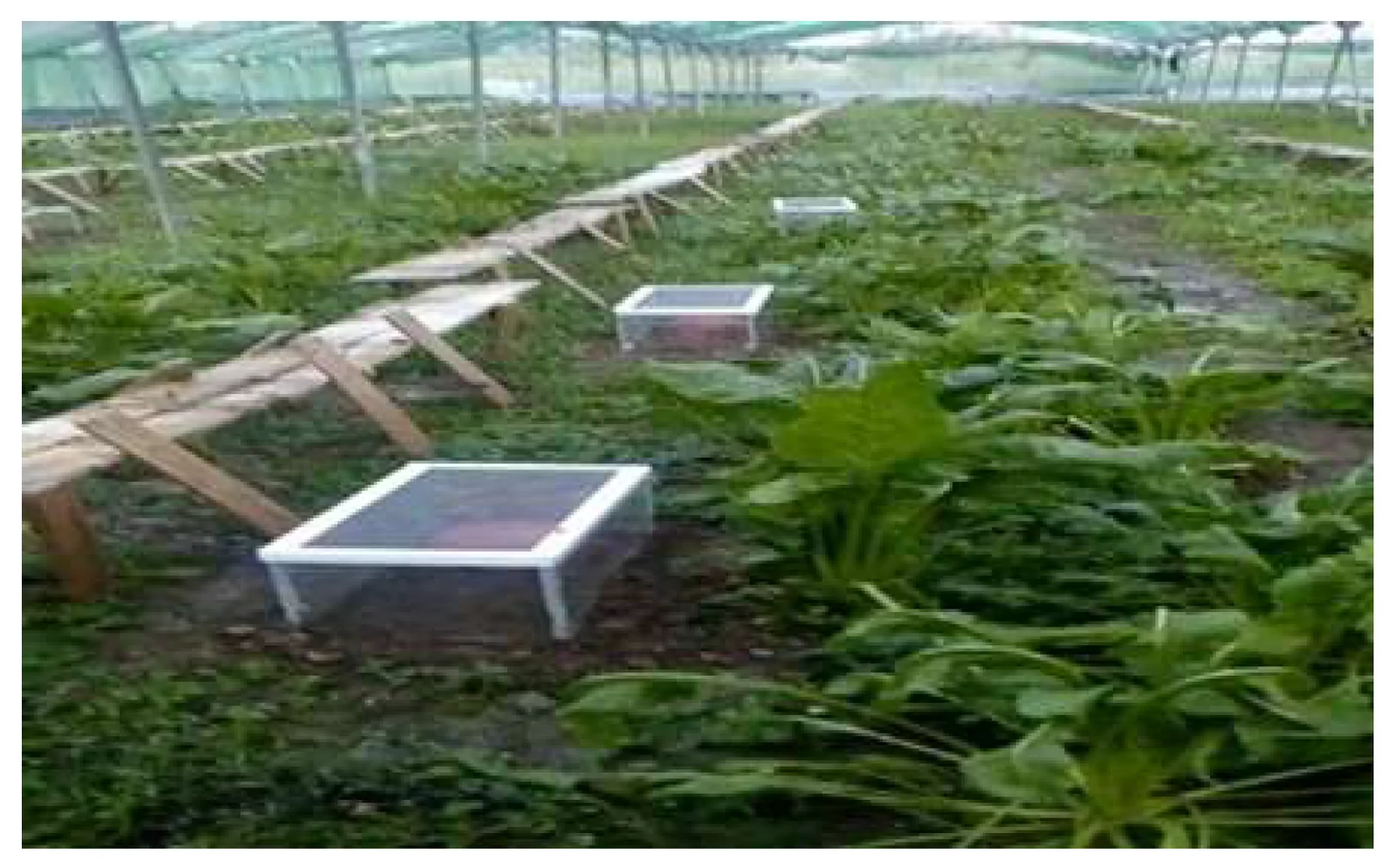
Experimental terraria placed within the greenhouse of the snail farming research facilities at the University of Thessaly.

A total of 500 *Cornu aspersum maximum* juveniles’ snails were provided by a local heliciculture farm and transferred to the experimental farming research facilities of the University of Thessaly (Volos, Greece) [28]. No ethical approval was required for using *Cornu aspersum maximum* in animal studies. However, the experiments were conducted according to the internal guidelines of the University of Thessaly, Greece. These guidelines are consistent with the national and European recommendations regarding the protection and welfare of laboratory/farm animals. The juveniles were acclimatized for 7 days before the formal experiment began. During the acclimation period, the snails were fed with the control diet. Snails were placed in custom-made experimental *terraria*. Each *terrarium* was positioned in a predefined area inside the greenhouse, where vegetation had been removed (Figure 1). *Terraria* were made from plexiglass and partially buried (2–3 cm deep) to enhance structural stability. Lids for the boxes were constructed from aluminum rods, over which shade netting was affixed using industrial-grade metal adhesive. Each lid was labeled with the corresponding treatment/replicate group code.

After the acclimation period, 270 juvenile snails were uniform in size, and they were randomly divided into three groups (90 snails per group). Each group had three replicates, for a total of 9 *terraria* (3 diets × 3 replicates). The initial average weight of the snails was 1.78 ± 0.07 g. Each terrarium contained 30 juvenile *Cornu aspersum maximum*. Temperature ranged from 14.1 to 27.4 °C and relative humidity from 55.4 to 86.8% throughout the 60-day feeding experiment. Diets were offered in equal amounts (20 g) to each terrarium every second day, resulting in a total of 30 feedings over the 60-day experimental period. The control group received standard feed without GM supplementation (Table 2), whereas the experimental groups were administered feeds supplemented with 7% and 14% GM flour respectively (Table 1). The feeding cycle lasted for 60 days. Feed consumption was recorded in every feeding by weighing feeds offered to and refused by snails. Feed intake (FI) was estimated in g by the average consumed feed throughout the experiment for all dietary groups. Feed intake index (FII) was then calculated in grams per day per snail (g/d/snail). On 0, 15, 30, and 45 days of the experiment, the average body weight (BW) of each dietary group was estimated. Final body weight (FBW) at day 60 and BW at day 0, 15, 30, and 45 of the snails were recorded using a precision balance (EMB 200-2, Kern & Sohn, Balingen, Germany) on the last day of the experiment. Growth performance (GP) for every group was measured by the average gained weight of the snails (FBW—initial body weight). The FI/GP ratio was also estimated.

### 2.3. Snail Sampling

At the end of the experiment, a total of 180 snails with approximately identical BWs were collected from (60 per dietary group, 20 per terrarium) and transferred to the laboratory. FBW of raw snails was measured in each specimen using a precision balance (EMB 200-2, Kern & Sohn, Balingen, Germany) with two decimal places. All animals were slaughtered, and their shells were removed. Proximate composition amounted to 30 snails per dietary group (CF0, CF7, and CF14). Another 27 snails (9 per dietary group) were used to evaluate fillet qualitative properties, and 15 snails were allocated for parasitological analysis (5 snails per dietary group). Also, fresh fillet samples of approximately 1 g were taken from 10 snails per dietary group and were put in Eppendorf tubes and stored at −80 °C for chemical analyses.

### 2.4. Parasitological Analysis

Fecal samples were examined using the Concentration McMaster technique, a standard procedure for quantifying the number of nematode eggs per gram of feces (EPG). This technique is slightly more complicated than the Simple McMaster Technique, but the sensitivity is better (20 EPG) [29].

Each snail was examined individually under a Carl Zeiss Light Microscopy (Carl Zeiss Ltd., Gottingen, Germany) to detect potential infections with nematode larvae. After 24 h, the shell was removed, and the fillet was separated and individually placed in a beaker. The mantle cavity and foot tissues were examined according to the method described by Valente et al., 2017 [30], followed by analysis of the internal organs as outlined by Franco-Acuña et al., 2009 [31].

### 2.5. Chemical Analysis

Dry matter content of the diets and snails’ meat was determined by oven (TS 8056; Termaks, Bergen, Norway) drying at 105 °C for 48 h, and the water content was determined gravimetrically [32]. Ash content (% *m*/*m*) was recorded in a water-free sample that was combusted in a muffle furnace (Nabertherm L9/12/C6, Lilienthal, Germany) by heating at 600 °C for 3 h, and the ash content was measured gravimetrically. Crude protein content was determined by the Kjeldahl method (N × 6.25; Behr Labor-Technik, Düsseldorf, Germany [32]. Crude fat content was determined by the Soxhlet method (SOXTHERM^®^ SOX416 macro; Gerhardt, Königswinter, Germany). Gross energy was determined by means of an adiabatic IKA oxygen bomb calorimeter (C7000, IKA C7000, Staufen, Germany). All measurements were carried out in triplicate, and the values were averaged.

Carbohydrate content of the diets and snails’ meat was determined using a digital refractometer (MRC, ref-85 portable Refractometer, Holon, Israel). The pH measurement of the diets and snails’ meat was performed using pH meter (pH60 DHS). Ten g of dried sample was homogenized with 90 mL distilled water. After 30 min of stirring, the pH measurement was conducted in the supernatant. All measurements were carried out in triplicate, and the values were averaged.

**Figure 2:**
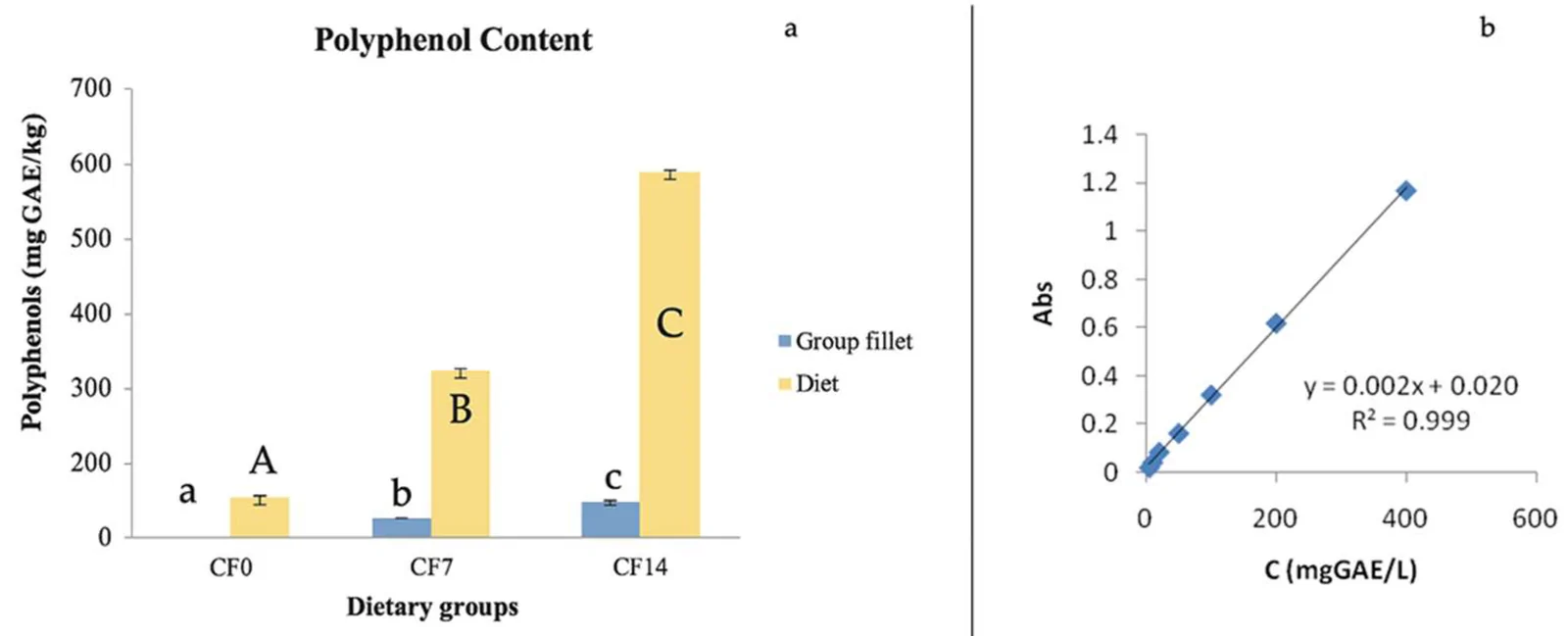
(**a**). Rates of total polyphenol content (mg GAE/kg) on the diets and the fillets of the three dietary groups (CF0, CF7, and CF14). Whiskers represent standard deviation (*n* = 3) and (a–c, A–C) indicate significant differences among experimental dietary groups (*p* < 0.05). (**b**). Calibration curve for different concentrations (C 5—400 mg GAE/L) of gallic acid.

The Total Polyphenol content of the diets and snails’ meat was determined using the Folin–Ciocalteu assay. A 0.5 g portion of homogenized sample was left in 5 mL of ethanol (Merck) overnight at room temperature. After the extraction, 5 min of centrifugation at 2000 rpm was used to separate the total polyphenol extract. Afterwards, 100 µL of the supernatant was added into 3.50 mL of ultrapure water (18.2 MΩ cm at 25 °C), and then 100 µL of the Folin–Ciocalteu reagent (Merck) were added and mixed well. After 2 min, 300 µL of a 20% (*w*/*v*) solution sodium carbonate (Chemlab) was added, and the samples were well mixed. Finally, the samples were left at room temperature for 2 h, and the absorbance was measured at 765 nm using a HACH DR 6000 UV–Vis spectrophotometer. Each sample was measured in triplicate, and results were expressed as mean concentrations in mg gallic acid equivalents (GAE) per kg of wet weight [33]. A calibration curve (Figure 2) was constructed using gallic acid standard solutions (Riedel-de Haën) ranging from 5 to 400 mg GAE/L. This range was selected to avoid time-consuming dilutions of the samples and to ensure the centroid standard solution of the calibration curve close to the content of the samples for achieving more accurate results. The limit of detection (LOD) was 17 mg GAE/kg. The LOD was calculated by multiplying the standard error of the intercept by 3.3 and dividing the calculated result by the slope. A similar procedure was followed for the calculation of the limit of quantification (LOQ), but the factor for intercept multiplication was equal to 10.

The Total Antioxidant Activity (TAA) of the diets and snails’ meat was evaluated using the DPPH assay. A 0.5 g portion of homogenized sample was left in 5 mL of ethanol (Merck) overnight at room temperature. After the extraction, 5 min of centrifugation at 2000 rpm was used to separate the total antioxidant extract. The extracts were vaporized and diluted to 5 mL of ethanol. The sample extracts at different concentrations (ranging from 200 mg/L to 5000 mg/L) were mixed with 0.4 mL of DPPH solution (80 mg/L FLUKA). The reaction mixtures were incubated at room temperature for 20 min, after which the absorbance was measured at 515 nm using a HACH DR 6000 UV–Vis spectrophotometer. The percentage of DPPH radical inhibition was calculated, and EC_50_ values were determined from the resulting inhibition curves (percentage inhibition vs. concentration), with all curves exhibiting high linearity (R^2^ > 0.995). The results were also expressed as the Antioxidant Activity Index (AAI) [33]. The AAI was calculated by dividing the DPPH concentration to the EC_50_ value.

### 2.6. Qualitative Properties of Snail Fillets

Nine fillets from each dietary group (CF0, CF7, and CF14) used for color and hardness assessment were subjected to the following processing stages: steaming in a steaming tunnel (at 100 °C for 5 min), boiling in water bath (at 100 °C for 20 min), removal of edible tissue (fillet and visceral mass) from shell, and separation of fillet according to Kougiagka et al., 2022b [34]. The weight (FW) was assessed using a precision balance (EMB 200-2, Kern & Sohn, Balingen, Germany), and the thickness (FT) of boiled fillets was measured by digital calipers (Powerfix Profi Digital Caliper, Germany resolution: 0.01). As edible portion (EP) was estimated, the percentage of boiled fillets weight compared to the weight of fresh snail.

The instrumental texture measurements were performed on four boiled fillets from each dietary group (CF0, CF7, and CF14), chosen from the nine fillets used for color assessment as texture profile analysis (TPA), at 5 °C after 75% compression using Admet texture analyzer eXpert 5601 (Admet Texture Analyzer eXpert 5601; AdMEt, Inc., Norwood, MA, USA) and cylindrical probe of 18.0 cm diameter with speed 100 mm/min. The texture attribute “hardness” is defined at the maximum force (Fmax) of the first compression (Fmax). For each group of four boiled fillets, average Fmax values were evaluated [23,34].

Color measurements (CP) were performed at 5 °C on the surface of the ventral region of boiled fillets using colorimeter (HunterLab MinScan XE Plus, Reston, VA, USA) in the CIELAB color space [35]. Lightness (L*), redness (a*), and yellowness (b*) were recorded per group. L* or lightness was expressed in a dark-to-light scale of 0 to 100. The parameters a* or redness shows green to red, and b* or yellowness represents blue to yellow, both on a scale from −60 to +60. Nine fillets from each dietary group (CF0, CF7, and CF14) divided into three groups of three snail fillets were used for color assessment. Three replicate measurements were obtained and averaged [34].

### 2.7. Statistical Analysis

Data for statistical analysis were evaluated for normal distribution by employing the Shapiro–Wilk test for normality and homogeneity of variance by employing Bartlett’s and Levene’s tests. Data were analyzed by one-way ANOVA followed by Tukey’s test to identify possible differences between sample means. Statistical analysis was performed with Jamovi Software (2.3.18) [36]. The level of statistical significance was set at *p* < 0.05.

## 3. Results

### 3.1. Production Performance

The FI and FII parameters presented (Table 3) higher amounts of value as the inclusion of GM was enhanced in the diets. Especially, FI’s value got higher as the enrichment percentage went up. GP did not indicate any significant difference for the three dietary groups. Statistical analysis shows that FII of CF0 differ significantly from the other diets. Also, in FI, it is indicated that every treatment shows differences between each other.

Although the dietary groups fed with GM consumed more feed than the control dietary group, their growth was not affected by it, resulting in the improved digestibility of diets containing GM (Table 3).

The biggest increase in BW (3.67 g) of almost all dietary groups was recorded from 30 d to 45 d, which matches an optimized feeding period (mid–late September) of the species. One-way ANOVA indicates significant difference (*p* < 0.001), between snails fed with CF0 and gastropods with GM diets (CF7, CF14). It is important to stress that until day 30, BW of snails in CF0 and CF7 was similar.

### 3.2. Parasitological Analysis

The prevalence of susceptible species of snails is an important factor in determining the density of molluscan infection with the nematode. In our experiment, there was no adult Nematoda parasite.

On the other hand, the results from Concentration Mcmaster technique were positive for every sample. Analytically, we found 2480 EPG in the first group (CF0) of snails, 700 EPG in second (CF7), and 700 in third where the snails were fed with CF14.

### 3.3. Chemical Analysis

The chemical composition of the three groups’ fillet did not indicate any significant difference based on the diet’s choice. The only difference regarding their nutritional composition was indicated by the percentage of CF14’s DM (24.14%) which was higher (*p* < 0.001) than CF0’s (21.35%) and CF7’s (21.14%) DM (Table 4).

The polyphenol content in the group’s fillet (Figure 2a,b) doubled with the increase of grape marks in the diets, depending, of course, on the percentage of that inclusion.

**Figure 3:**
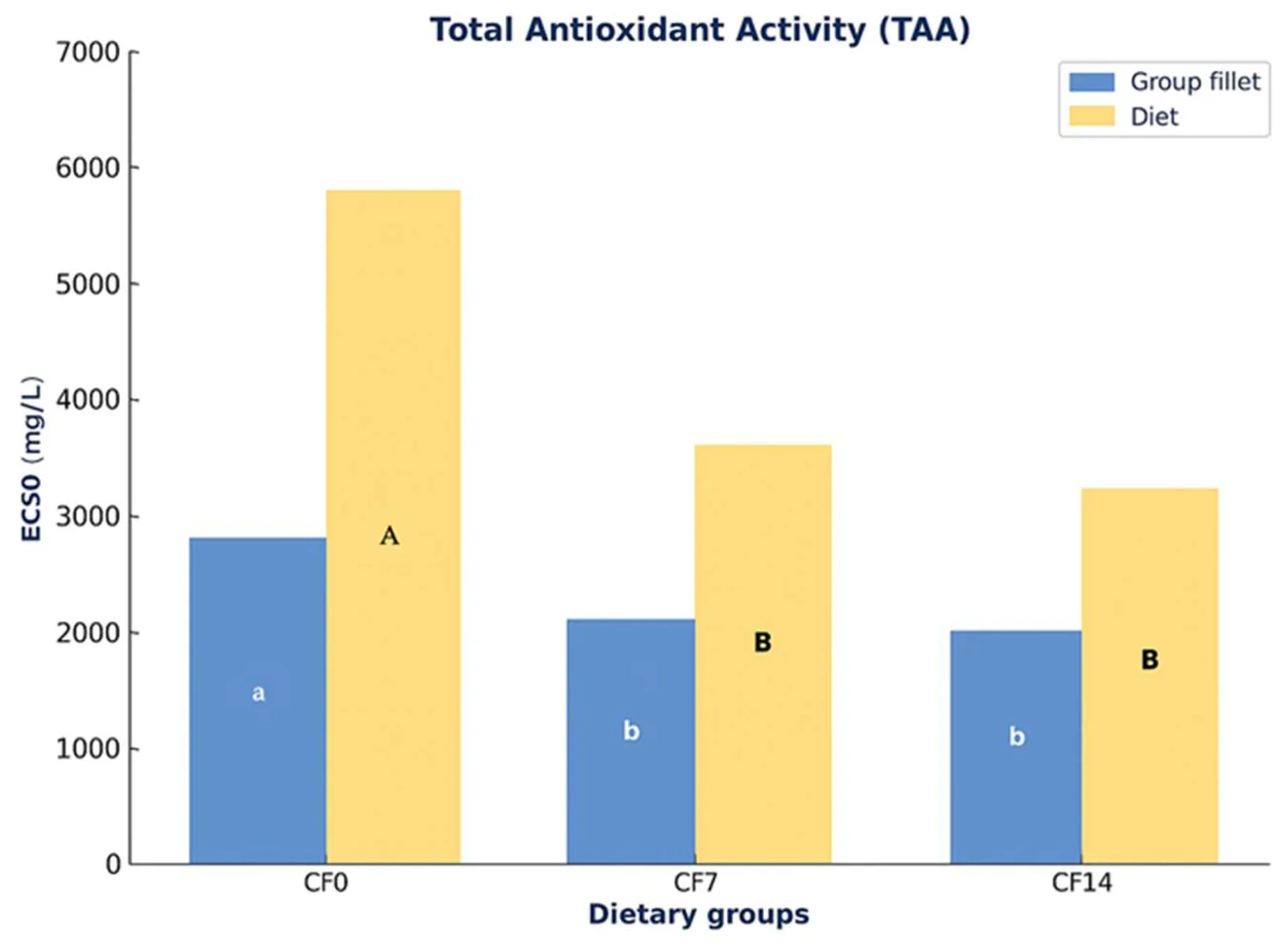
Effect of the inclusion of GM on Total Antioxidant Activity (TAA) on the diets and the fillets of the three dietary groups (CF0, CF7 and CF14), measured in mg/L of EC_50_. (a,b,A,B) indicate significant differences among experimental dietary groups (*p* < 0.05).

As much as TAA is concerned, an improved antioxidant activity was detected in both diets and the group’s fillet (Figure 3), based on the inclusion of GM in them.

**Figure 4:**
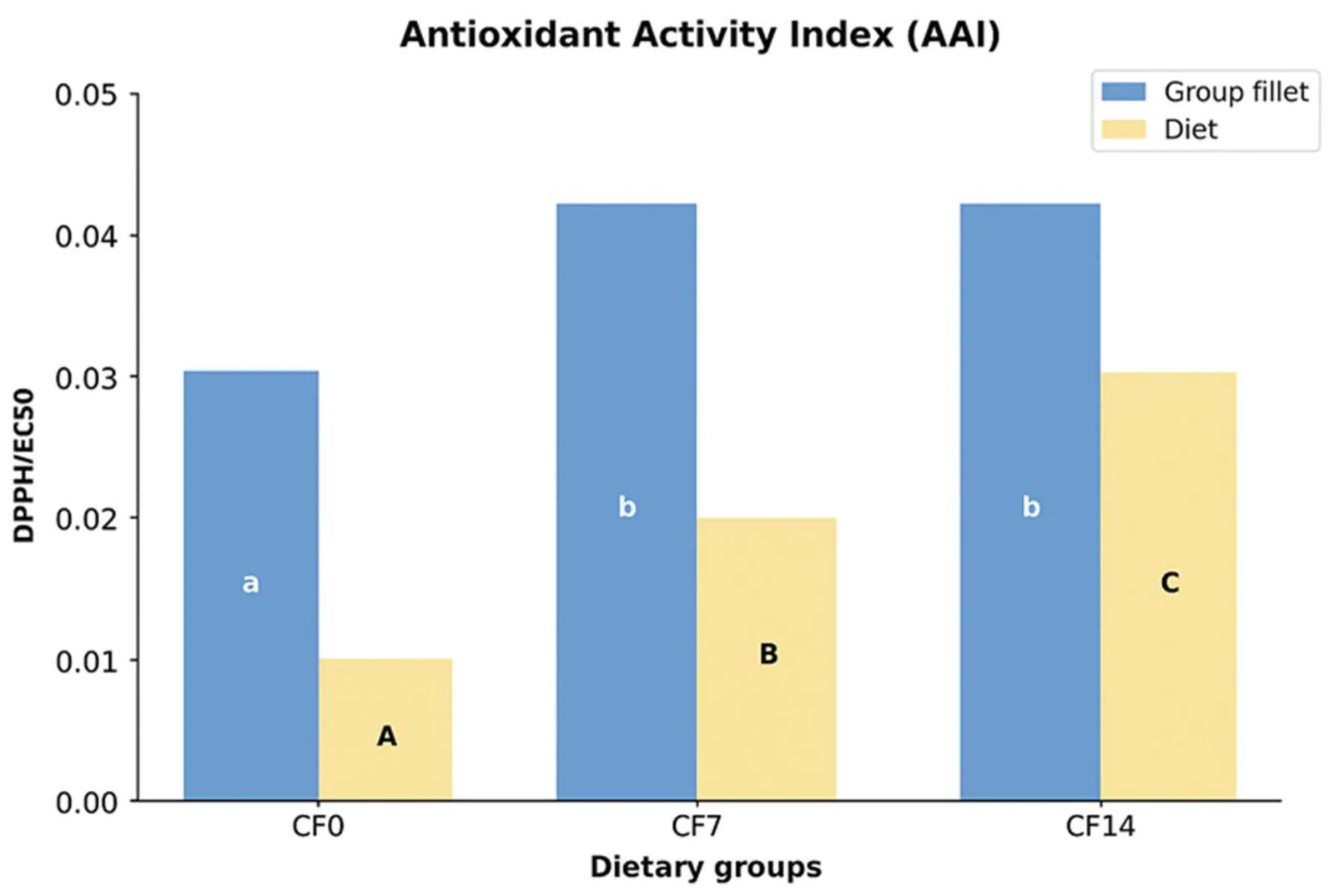
Effect of the inclusion of GM on Antioxidant Activity Index (AAI) on diets and the fillets of the three dietary groups (CF0, CF7, and CF14), calculated by dividing the DPPH concentration by the EC_50_ value. (a,b, A–C) indicate significant differences among experimental dietary groups (*p* < 0.05).

On the other hand, AAI in the diets got higher regarding the inclusion of GM in them, but AAI in the group’s fillet got lower only for CF0, compared to CF7 and CF14, which was identical (Figure 4), indicating that GM improved AAI regardless of the percentage of inclusion in the diets.

### 3.4. Qualitative Properties of Fillets

According to Table 5, fillets of CF7 group presented the highest value of fillet weight (1.37 ± 0.31 g), while no statistically significant differences were reported for the weight of enriched and non-enriched snail fillets. Fillet thickness decreased after 14% feed supplementation (5.43 ± 1.07 mm). Also, feed supplementation did not affect the percentage of edible portions, which ranged from 18.81 to 20.25%. The enrichment of 7% and 14% led to same values of tenderness as fillets from enriched groups had more tender texture (6.14 and 6.02 N respectively) than the control group (9.04 N). Finally, color parameters did not present statistically different values among groups, while diet supplementation led to lower values of L* lightness and a* redness, apart from b*yellowness, which increased in the snail fillets fed with CF14.

## 4. Discussion

In recent years, studies have explored the use of GM in poultry and pig diets [37,38], primarily due to the high content of polyunsaturated fatty acids in their meat, which makes it more susceptible to oxidative degradation during storage. Enriching the diet of *Cornu aspersum maximum* can improve growth rates, enhance nutritional quality for human consumption, and increase overall farming efficiency. Notably, this was the first study that used GM as a feed component in heliciculture, highlighting its potential as an alternative and sustainable dietary source. *Cornu aspersum maximum* is one of the main farmed species, whose morphology and genetic profile has been extensively studied [39], and it is necessary to investigate all the aspects of diet enrichment to enhance food quality.

### 4.1. Production Results

In the present study, the increase in FI observed in the CF14 group (17.82 ± 1.33 g/snail), along with the FII in both enriched groups (0.11 ± 0.01 g/day/snail, *p* < 0.05), were statistically significant compared to the control. These findings align with the results of Ragni et al., 2013 [26], who reported that the addition of grape seed flour to diets for fattening lambs led to increased feed consumption, suggesting that the supplement enhances palatability. Furthermore, Derbali et al. (2024) [40] demonstrated that in rabbits, dietary supplementation with grape pomace led to an increase in FI, a finding that is consistent with the outcomes of the present study. Similarly, Massaro et al. [41] demonstrated that the inclusion of 0–30% grape pomace silage in lamb diets did not negatively affect GP. The comparison is drawn with mammals which have been extensively investigated, owing to the absence of relevant studies on snails or other farmed invertebrates. In a recent study, Bullon et al. (2025) [10] demonstrated through their experiment on farmed abalone (a species of marine mollusk) that the inclusion of GM in the diet may be associated with enhanced growth performance, indicating its potential as a sustainable feed additive. This is consistent with our results: although a slight decline in GP was observed in the CF7 and CF14 groups, the difference was not statistically significant. The FI/GP ratio was also higher in the enrichment groups compared to the control, likely due to the increased FI.

In our experiment, the farmed snails reached their marketable size within two months, increasing their initial weight tenfold. FBW of fresh snails was highest in the control group; however, the weight of heat-treated fillets did not differ significantly among groups. The edible portion was also similar across treatments, ranging from 18.11% to 20.25%, with no statistically significant differences. Overall, these findings are consistent with previous research [42], which reported that GM, as a cost-effective dietary supplement, can maintain animal productivity and health in sheep and farm animals. In Greece, the use of poultry feed is widespread in heliciculture [12], and was selected as the control diet in our experiment. GM is generally considered an agro-industrial by-product and is often available at low cost. Its inclusion in the snail diet could potentially reduce feed costs, especially if it replaces more expensive protein or fiber sources.

### 4.2. Animal Health

The are only a few studies that have applied the McMaster technique for quantifying nematode EPG in farmed snail feces. Apostolou et al., 2019 [43], in a two-year study, reported notably high mean EPG values: 3457.33 in 2017 and 2535.55 in 2018. However, in more recent research, Apostolou et al., 2023 [28] recorded only 1060 EPG in feces of farmed snails. In our study, the highest EPG observed was 2480 in the commercial diet group (comparable to Apostolou’s second year), while significantly lower values (as low as 700 EPG) were recorded in the GM-supplemented groups. Furthermore, although Apostolou et al., 2021 [12] reported the presence of adult parasites in 22.7% of snail farms examined in the first year and 38.8% in the second, no adult parasites were detected in any of the snails from our study. This finding suggests that GM may possess antiparasitic properties that help snails resist infection. This antiparasitic activity of grape extract is attributed to the polyphenols it contains. This hypothesis is supported by other studies, such as that by Mena et al., 2025 [44], which demonstrated that grape pomace supplementation led to reduced fecal egg counts and decreased nematode fecundity in lamps with the caveat that direct comparison between invertebrates and mammals should be made cautiously. Furthermore, Rama et al. (2021) [45] demonstrated that wine natural extract has been shown to be effective against parasites affecting humans, animals, and plants. Moreover, the study by Soares et al. (2018) [46] demonstrated that grape extract exhibits in vitro anthelmintic activity (ovicidal and larvicidal), suggesting that, in addition to its nutritional potential, grape seed by-products may also serve as an ally in the control of gastrointestinal nematodes in sheep.

### 4.3. Product Quality

Additionally, the results demonstrated improved antioxidant activity in both GM-enriched diets and the corresponding snail fillets. AAI values were higher in the enriched groups, reflecting the inclusion of GM. The lower AAI observed in the control group indicates that GM supplementation enhanced antioxidant capacity, regardless of the inclusion level. Similar findings have been reported in previous studies: Hao et al., 2015, Zheng et al., 2025, Tian et al., 2023, and Proca et al., 2024 demonstrated increased antioxidant activity following grape supplementation in pigs [47,48,49,50]; comparable results were also found in broiler chickens by Makris et al., 2007 [9]. It is worth noting that these findings should be interpreted with the limitation that they refer to studies in vertebrates and not in invertebrates, specifically mollusks, since our work represents the first report on snail meat.

According to the hardness assessment, diet supplementation with GM resulted in more tender snail fillets, regardless of the inclusion level. Fillets from the control group exhibited the highest hardness value (9.04 N), while those from the CF7 and CF14 groups showed significantly lower values—6.14 N and 6.02 N, respectively. In contrast, Kougiagka et al., 2022b [34] reported greater tenderness in *Cornu aspersum maximum* snail fillets, with values averaging 3.14 ± 0.38 N, as they used cylindrical parts from the middle-posterior region of boiled fillets for their measurements. The cylindrical part consists solely of muscle tissue, in contrast to the entire fillet used in this study, which integrates components from several body systems. Moreover, heat treatment contributes to a more tender texture by inducing significant histological changes in snail muscle tissue, including the denaturation of muscle proteins and the breakdown of collagen fibers. Similarly, Cimmino et al., 2018 [51] found no significant differences in shear force in Saanen goat kid meat between animals fed a standard diet and those receiving a polyphenol-enriched diet. The comparison is drawn with mammalian meat, which has been extensively investigated, owing to the absence of relevant studies on snails or other edible invertebrates’ meat.

The color of meat is a complex attribute influenced by various factors, and while adding GM to animal feed can potentially affect this characteristic, the outcomes vary depending on multiple variables. The key to maintaining desirable meat color lies in finding the right balance in GM inclusion and managing the overall diet to ensure that marbling and fat content align with consumer preferences [52]. In terms of color parameters, fillets from snails fed the control diet had the highest values for L* (lightness) and a* (redness). Notably, except for the 7% supplementation level, higher levels of GM in the diet were associated with an increase in fillet yellowness (b*). The reduction in the a* parameter in the CF7 and CF14 groups may be attributed to decreased oxidative stability of fat due to the increased PUFA content, as suggested by Bertol et al., 2017 [53]. However, no statistically significant differences were observed in the overall color parameters, consistent with findings from other species fed with supplemented diets [26].

## 5. Conclusions

The results demonstrated that GM supplementation (7% and 14%) can be included in the diet of farmed snails, resulting in a slight increase in FI without compromising growth performance. These findings suggest that GM could be valuable feed additive to improve animal health and productivity. However, the higher number of EPG in the CF0 group compared to CF7 and CF14 suggests that the inclusion of GM in the diet reduces EPG in the feces of snails fed the supplemented diets. Feed enrichment improved the tenderness of heat-treated fillets, with no changes in coloring, which can enhance consumer acceptability. Specifically, 14% supplementation resulted in more tender fillets without reducing fillet weight compared to the control group. However, both enriched groups showed improved results in qualitative characteristics, parasitological analysis, polyphenol content, and TAA when compared to control group.

Enriched diet may lead to the production of functional food which fulfils consumers’ demands for high-quality products. Additionally, replacement of one part of commercial snail feed with GM reduces fixed costs in snail farming, especially if it replaces more expensive protein or fiber sources. Notably, this was the first study that used GM as a feed component in heliciculture, highlighting its potential as an alternative and sustainable dietary source. Further research is necessary to evaluate different levels of polyphenols and to better understand the role these compounds may have in production performance and qualitative properties of farmed snails.

## Figures and Tables

**Table 1:** Composition of GM flour and the three experimental diets (CF0, CF7, and CF14). Values are expressed on a dry matter basis and include dry matter content (DM), crude protein (CP), crude fat (CF), ash content, and gross energy (GE, in KJ/g).

Chemical Analysis	GM	CF0	CF7	CF14
DM (%)	83	87.9	87.6	87.5
CP (%)	13.78	20.33	19.08	18.64
CF (%)	7.05	4.03	4.01	3.98
GE (KJ/g)	21.11	16.39	16.76	16.86
Ash (%)	3.99	6.39	6.35	6.65

**Table 2:** Proximate chemical composition of the control diet (starter poultry feed), as provided by the manufacturer. Values are expressed on a dry matter basis and include dry matter content (DM), crude protein (CP), crude fat (CF), crude fiber, ash content, gross energy (GE, in KJ/g), and vitamins and minerals (per kg).

Chemical Analysis	(%)	Vitamins and Minerals	(Additives per kg)
DM (%)	82	BIT A	12,000,000 IU
CP (%)	21	BIT D3	4,000,000 IU
CF (%)	3.7	BIT E	100,000 mg
Crude fiber (%)	4.5	BIT K3	9000 mg
Ash (%)	5.6	BIT B1	3000 mg
Calcium (%)	1.2	BIT B2	7000 mg
Total phosphorus (%)	0.7	BIT B6	6000 mg
Lysine (%)	1.1	BIT B12	35 mg
Methionine (%)	0.48	Biotin	200 mg
Sodium (%)	0.18	Iron	50,000 mg
Carbohydrates (%)	12.8	Iodine	1500 mg
GE (KJ/g)	17.2	Cobalt	250 mg
		Copper	20,000 mg
		Manganese	150,000 mg
		Zinc	100,000 mg
		Selenium	350 mg
		Folic Acid	1500 mg
		Pantothenic Acid	15,000 mg
		Nicotinic Acid	70,000 mg
		Vit. Stay-C 35%	50,000 mg

**Table 3:** Mean values (±standard deviation) of production performance parameters of snails fed with three different diets (CF0, CF7, and CF14) over a two-month experimental period. FII: Feed intake index (g/day/snail); FI: Total feed intake per snail (g), GP: Growth performance (g); FI/GP: Feed conversion ratio; BW: Snail body weight (g) at different time points (day 0, 15, 30, 45); FBW: Final body weight (g).

Production Performance	CF0	CF7	CF14	*p*-Value
FII (g/d/snail)	0.10 ± 0.02 ^A^	0.11 ± 0.01 ^B^	0.11 ± 0.01 ^B^	<0.001
FI * (g/snail)	16.75 ± 1.56 ^A^	17.34 ± 1.20 ^B^	17.82 ± 1.33 ^B^	<0.05
GP (g)	9.93 ± 3,84	9.06 ± 3.50	9.21 ± 3.75	ns
FI/GP	1.69	1.91	1.93	ns
BW at d 0 (g)	1.79 ± 0.03	1.79 ± 0.03	1.8 ± 0.04	ns
BW at d 15 (g)	3.19 ± 0.29 ^A^	3.18 ± 0.08 ^A^	2.84 ± 0.15 ^B^	<0.001
BW at d 30 (g)	6.69 ± 0.78 ^A^	6.19 ± 0.48 ^B^	6.19 ± 0.53 ^B^	<0.001
BW at d 45 (g)	10.31 ± 0.75 ^A^	9.77 ± 0.92 ^B^	10.00 ± 0.68 ^A,B^	<0.001
FBW at d 60 (g)	11.72 ± 0.61 ^A^	10.86 ± 0.55 ^B^	11.03 ± 0.63 ^B^	<0.001

* Feed consumed with the CaCO_3_ (ad libidum) supplementation. Values in each row that do not share the same superscript letter indicate statistically significant differences (*p* < 0.05, *p* < 0.001). “ns” denotes non-significant differences.

**Table 4:** Nutritional composition of fillets from the three experimental dietary groups (CF0, CF7, and CF14). Values are expressed on a wet weight basis and include dry matter (DM), crude protein (CP), crude fat (CF), carbohydrate content (CH, in Brix units), ash content, gross energy (GE, in KJ/g), and pH values.

Nutritional Composition of Fillets	CF0	CF7	CF14	*p* Value
DM (%)	21.14 ^A^	21.35 ^A^	24.14 ^B^	<0.001
CP (%)	13.91	13.23	13.06	ns
CF (%)	0.62	0.58	0.55	ns
CH (Brix)	2.4	2.1	2.2	ns
Ash (%)	6.46	6.67	6.84	ns
GE (KJ/g)	20.95	20.57	20.52	ns
pH	8.68	8.72	8.53	ns

Values in each row that do not share the same superscript letter indicate statistically significant differences (*p* < 0.05, *p* < 0.001). “ns” denotes non-significant differences.

**Table 5:** Mean values (± standard deviation) of qualitative parameters of snail fillets across three dietary groups (CF0, CF7, and CF14). FW: Fillet weight (g), FT: Fillet thickness (mm), EP: Edible portion (%, calculated as the ratio of fillet weight to total body weight); HV: Hardness (N), CP: Color parameters [L* (lightness), a* (redness), b* (yellowness)]. Mean values of FW, FT, and EP are based on 10 fillets per group, HV on 4 fillets, and CP on 3 fillets.

Qualitative Properties of Fillets	CF0	CF7	CF14	*p* Value
FW (g)	1.27 ± 0.44	1.37 ± 0.31	1.29 ± 0.41	ns
FT (mm)	6.63 ± 1.04 ^A^	6.64 ± 0.93 ^A^	5.43 ± 1.07 ^Β^	*p* < 0.05
EP (%)	18.11 ± 2.74	20.25 ± 4.72	19.04 ± 4.39	ns
HV (N)	9.04 ± 1.19 ^A^	6.14 ± 1.06 ^B^	6.02 ± 0.58 ^B^	*p* < 0.01
CP	L* 55.83 ± 3.39	54.66 ± 6.53	54.62 ± 1.26	ns
	a* 3.08 ± 0.89	2.77 ± 0.54	2.53 ± 0.75	ns
	b* 12.00 ± 2.05	11.13 ± 1.87	13.03 ± 3.14	ns

Values in each row that do not share the same superscript letter indicate statistically significant differences (*p* < 0.05, *p* < 0.01). “ns” denotes non-significant differences.

## Data Availability

The original contributions presented in this study are included in the article. Further inquiries can be directed to the corresponding author.

## Abbreviations

The following abbreviations are used in this manuscript:

GM	Grape Marc
FI	Feed Intake per snail (g)
FII	Feed Intake Index(g/day/snail)
GP	Growth Performance
EPG	number of nematode Eggs Per Gram of feces
AAI	Antioxidant Activity Index
TAA	Total Antioxidant Activity
GAE	Gallic Acid Equivalents
TPA	Texture Profile Analysis
